# Supplementary material for: Neural network interpolation of exchange-correlation functional
Source: Sci Rep. 2020 May 14;10:8000. doi: 10.1038/s41598-020-64619-8 (PMC7224278; doi:10.1038/s41598-020-64619-8)
Supplement: Supplementary file 1 — Supplementary Information. [file 41598_2020_64619_MOESM1_ESM.pdf]

# Supplementary information

# Neural network interpolation of exchange-correlation functional

Alexander Ryabov[1,2], Iskander Akhatov[1], Petr Zhilyaev[*1]

1
Center for Design, Manufacturing and Materials, Skolkovo Institute of Science and Technology, Skolkovo Innovation Center, Building 3, Moscow, 143026, Russia; Email: p.zhilyaev@skoltech.ru

2
Moscow Institute of Physics and Technology (State University), Institutskiy per. 9, Dolgoprudny, Moscow Region 141700, Russia

# S1

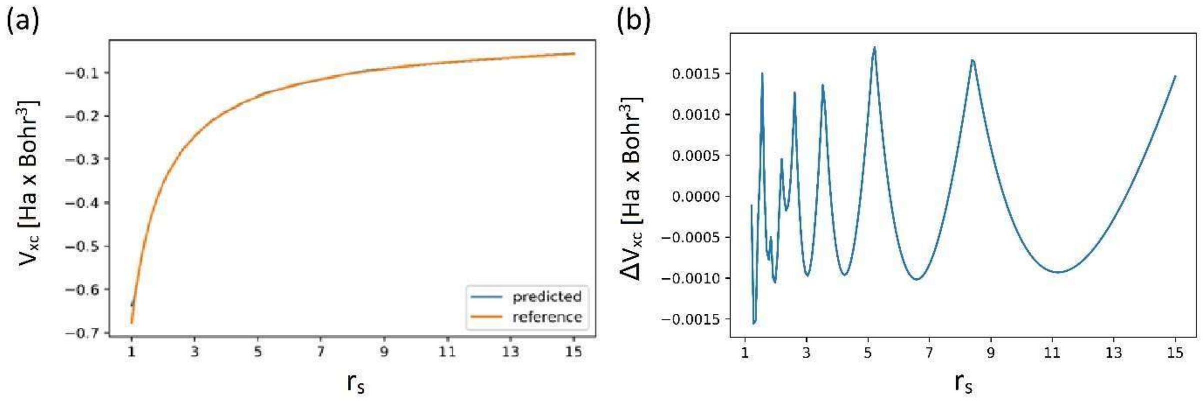


Figure S1. (a) LDA exchange-correlation potential versus $r_s$. Blue line – exchange-correlation potential obtained from NN, orange line – reference analytical LDA exchange-correlation potential. (b) Difference between exchange-correlation obtained from NN and reference analytical LDA exchange-correlation potential.

# S2

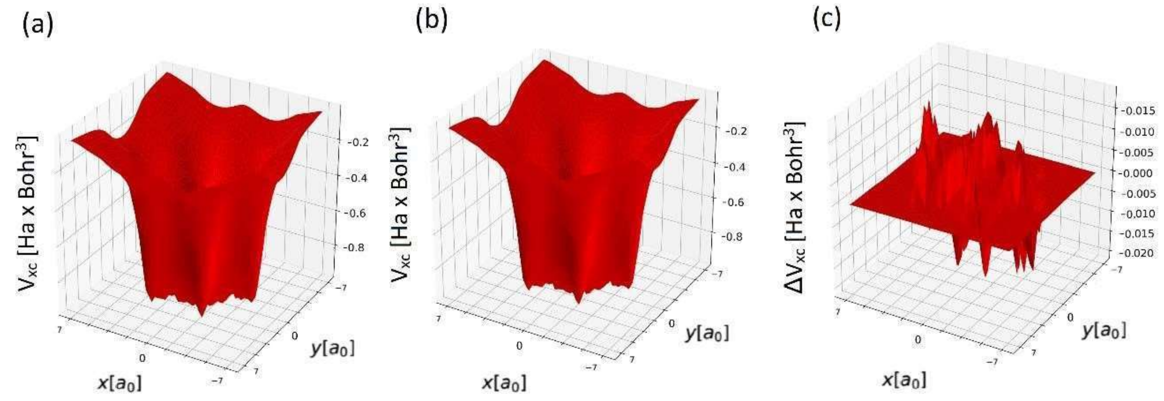


Figure S2. (a) Slice of LDA exchange-correlation initial potential for benzene at z = 0. (b) Slice at z = 0 of LDA exchange-correlation potential for 5x5x5 density cube rotated by 90 degrees along z axis passing through the centre of the cube. (c) Difference between slices specified in (a) and (b)
